# Books and Pamphlets Received

**Published:** 1884-09-20

**Authors:** 


### BOOKS AND PAMPHLETS RECEIVED.

*Diseases of the Throat and Nose*, including the Pharynx, Larynx, Trachea, Œsophagus, Nose and Naso-Pharynx, by Morell Mackenzie, M.D., London, Consulting Physician to the Hospital for Diseases of the Throat, Lecturer on Diseases of the Throat at the London Hospital, Medical College, and Corresponding Member of the Imperial Royal Society of Physicians of Vienna. Vol. II. Diseases of the Œsophagus, Nose and Naso Pharynx, with Index of Authors and Formulæ for Topical Remedies. Illustrated. Philadelphia: P. Blakiston, Son & Co., 1012 Walnut St. 1884.

Upon examination, we find this a most admirable work upon the important subjects treated. It is able, full and satisfactory, containing five hundred and fifty large octavo pages.

Affections of the nasal passages are becoming more and more numerous, and the treatment in a large proportion of cases is very unsatisfactory with most physicians, and especially with the ordinary practitioner, who, as a rule, has neither the knowledge nor the appliances for the proper treatment of such cases.

*Annual Report* of the Board of Regents of the Smithsonian Institute, showing the Operation, Expenditures and Condition of the Institution for the year 1883. Washington, D. C. 1884.

A Report, a work of about eight hundred pages, comes out annually from this Institution, and is always a work of much interest, especially to the scientific reader, as it contains a Scientific Record of facts, discoveries, etc., in various departments, including papers pertaining to ANTROPOLOGY, etc.

*Transactions of the Mississippi State Medical Association* at its Seventeenth Annual Session, held at West Point, April 2d, 3d and 4th, 1884.

A book of one hundred and ninety octavo pages, neatly gotten up.

Quite a number of able and interesting papers were presented at the Society, which we have not space to refer to in detail.

The officers elect for the year are the following:

President—D. L. Phares, M.D., Starkville.

Vice Presidents—J. B. Gresham, M.D., West Point; W. A. Taylor, M.D., Boonville.

Recording Secretary—W. E. Todd, M.D., Clinton.

Assistant Secretary—N. L. Clark, M.D., Hickory.

Corresponding Secretary—M. S. Croft, M.D., Jackson.

Treasurer—Jno. F. Hunter, M.D., Jackson.

